# Sustainable Fertilization with Ramial Chipped Wood Enhances Antioxidant Profiles in Tomato Varieties: An Untargeted Metabolomics Approach

**DOI:** 10.3390/antiox14111330

**Published:** 2025-11-05

**Authors:** Mohamed M. Abuhabib, Clara Abarca-Rivas, Julián Lozano-Castellón, Anna Vallverdú-Queralt, Johana González-Coria, Sebastian T. Soukup, Rosa M. Lamuela-Raventós, Maria Pérez, Joan Romanyà

**Affiliations:** 1 Polyphenol Research Group, Department of Nutrition, Food Science and Gastronomy, Faculty of Pharmacy and Food Sciences, University of Barcelona, 08028 Barcelona, Spain; mohamed.abuhabib@ub.edu (M.M.A.); claraabarca@ub.edu (C.A.-R.); jullozcas@gmail.com (J.L.-C.); avallverdu@ub.edu (A.V.-Q.); lamuela@ub.edu (R.M.L.-R.); 2 Institute of Nutrition and Food Safety (INSA-UB), University of Barcelona, 08028 Barcelona, Spain; jgonzalezco@ub.edu; 3 CIBER Physiopathology of Obesity and Nutrition (CIBEROBN), Institute of Health Carlos III, 28029 Madrid, Spain; 4 Department of Biology, Health and the Environment, Faculty of Pharmacy and Food Sciences, University of Barcelona, 08028 Barcelona, Spain; 5 Department of Safety and Quality of Fruit and Vegetables, Max Rubner-Institut (MRI)—Federal Research Institute of Nutrition and Food, 76131 Karlsruhe, Germany

**Keywords:** organic farming, woody residues, *Solanum lycopersicum*, metabolomic profiling, antioxidant compounds, phenolics

## Abstract

Tomatoes are among the most consumed vegetables within the Mediterranean diet, recognized as one of the healthiest dietary patterns in the world. This study evaluated the effects of four fertilization treatments on the antioxidant and metabolic profiles of four local tomato varieties: Cornabel (V1), Cuban Pepper (V2), Corno Andino (V3), and Roli Rosa (V4). Treatment 1 (T1) used 1.28 kg/m^2^ of commercial woody compost (C/N ratio 13), while Treatment 2 (T2) served as the control, initially without fertilization. Treatments 3 and 4 (T3, T4) incorporated ramial chipped wood (RCW) at 15 kg/m^2^ and 7.5 kg/m^2^, respectively, without tillage. Each treatment × variety combination included four biological replicates (*n* = 4). Untargeted metabolomic profiling via UHPLC-QToF and statistical analyses identified 163 compounds, 37 of which showed significant varietal differences (*p* < 0.05). The flavonoid eriodictyol was more abundant in the Pebroter varieties (V1, V2, V3), whereas luteolin-8-*O*-glucoside (orientin) predominated in V4, likely due to differential expression of biosynthetic genes. RCW (T3) yielded higher levels of antioxidant compounds like phenolic acids, and flavonoids compared to compost and control; however, no dose–response was observed using different doses of RCW (T3 and T4). These findings demonstrate that RCW may modulate the antioxidant metabolite profile of tomatoes, highlighting its potential as a sustainable fertilization strategy for enhancing antioxidant compounds.

## 1. Introduction

Tomato (*Solanum lycopersicum* L.) is one of the most widely consumed fresh vegetables worldwide. According to the Food and Agriculture Organization (FAO), approximately 170 million tons of fresh and processed tomatoes are produced globally each year, cultivated on approximately 5 million hectares of farmland [1]. Tomatoes are a key component of the Mediterranean diet, considered one of the healthiest dietary patterns and recognized for its role in preventing chronic diseases [2,3], and represent a major dietary source of bioactive compounds with strong antioxidant properties. They are particularly rich in carotenoids, such as lycopene and β-carotene, as well as polyphenols, flavonoids, and ascorbic acid, all of which contribute to their high antioxidant activity [4]. These compounds play a crucial role in neutralizing reactive oxygen species, thereby reducing oxidative stress and supporting the prevention of chronic diseases, including cardiovascular disorders and certain types of cancer [2,5]. Importantly, the antioxidant potential of tomatoes is determined not only by individual metabolites but also by the synergistic interactions among them, which can be effectively captured using metabolomic approaches [6,7]. Therefore, evaluating how agricultural practices, such as sustainable fertilization, impact the antioxidant profile of tomatoes is important both for promoting sustainability and for enhancing their antioxidant content.

Tomato yield and quality depend strongly on soil conditions and fertilization practices [8,9]. Under the pressures of climate change, European agricultural soils face declining quality, and there is an urgent need to adapt agricultural practices to achieve the United Nations Sustainable Development Goals for 2030 [10]. Approximately half of the soils in Europe are estimated to have a low content of organic matter [11], which significantly reduces their capacity to retain water and essential nutrients, or to store carbon. The Mediterranean region is particularly susceptible to soil degradation [12], exhibiting the lowest levels of soil organic matter [13] and highest erosion rates in the EU [14], as well as severe salinization problems [15]. Additionally, overuse of organic or chemical fertilizers has led to excessive nitrogen and phosphorus levels in soils [16], contributing to environmental degradation and raising public health concerns [17]. In response, sustainable agricultural practices—such as organic and regenerative farming—have been developed as viable solutions to reduce the adverse effects of synthetic fertilizers and pesticides on human and environmental health [18].

Organic and sustainable farming methods include the use of cover crops, green manures, and ramial wood by-products (i.e., chipped branches without foliage) as exogenous, plant-derived organic amendments [19]. These inputs contribute to soil organic matter, which not only enhances nutrient availability but also promotes root development, soil biodiversity, and microbial activity [20]. Studies have shown that organic fertilization can match the effectiveness of inorganic fertilizers in improving crop yield and quality [21], reinforcing its central role in organic farming [22]. Moreover, by simultaneously meeting the nutritional needs of plants and suppressing pest populations, organic fertilizers play a dual role in sustainable agriculture. Common sources include composted livestock manures, plant residues, and certain types of industrial waste [23].

Wood by-products, in particular, have been shown to enhance soil physicochemical and biological properties, including organic matter content, microbial activity, water retention, and nutrient availability [24]. Although the high C/N ratio of this type of fertilizer can initially lead to nitrogen immobilization, and temporarily reduce crop growth, improved yields have been observed during subsequent cropping. This delayed benefit is attributed to increased nitrogen release following the prolonged decomposition of the ramial woody residues in the soil [25]. Additionally, the mobilization of nutrients retained in the soil, such as nitrogen and phosphorus, largely depends on plant requirements and interactions with soil organisms [26]. Moreover, Pérez-Llorca et al. (2025) found that RCW applications increased soil organic carbon and microbial biomass [27], indicating improved soil health and nutrient cycling [28]. These findings emphasize the importance of understanding the effects of RCW on crop quality, particularly in terms of antioxidant composition, which remains underexplored.

Although ramial chipped wood (RCW) has been studied as a fertilizer in tomato cultivation, most research has focused on its effects on yield, with limited attention given to its impact on the phytochemical and antioxidant composition of the fruit [25]. Antioxidant compounds such as phenolics, vitamin C, and carotenoids are fundamental to the nutritional quality and health-promoting properties of tomatoes, and their levels may be strongly influenced by agricultural practices. To distinguish these complex metabolic changes, untargeted metabolomics provides a comprehensive approach for profiling antioxidant-related metabolites and their variation across treatments. Based on previous findings, we hypothesize that the incorporation of RCW may initially lead to a slight reduction in tomato yield during the first growing season due to temporary nitrogen immobilization [25], while simultaneously inducing moderate nutritional stress capable of modifying the metabolomic profile of the fruit. Therefore, this study evaluates both the agronomic performance and the antioxidant-related phytochemical profiles of four widely consumed traditional local tomato varieties: Cornabel, Cuban Pepper, Corno Andino, and Roli Rosa. Using a controlled experimental design, we compared the effects of RCW application at two different concentrations (15 kg/m^2^ and 7.5 kg/m^2^) with those of a standard commercial organic compost and nitrogen-rich organic pellets.

## 2. Materials and Methods

### 2.1. Chemicals and Reagents

Water, acetonitrile and methanol (all LC-MS grade, LiChrosolv^®^, Merck, Darmstadt, Germany) were purchased from VWR (Bruchsal, Germany). 98–Formic acid was obtained from Honeywell (Morristown, NJ, USA). Ammonium formate ≥99% was purchased from Sigma-Aldrich (Merck, Darmstadt, Germany).

### 2.2. Field Experiment and Plant Material

The study was carried out at Masia Cal Notari, an organic horticultural farm located in the peri-urban area of Barcelona (Sant Boi de Llobregat, Barcelona, Spain, 41°19′4.8″ N, 2°3′3.6″ E, 2 m.a.s.l.). The soil is classified as silty clay loam (sand: clay ratio 5.5:28.5) with 36.17% calcium carbonate, and the area has a typical Mediterranean climate. During the experimental period, seasonal mean temperatures were recorded as follows: May 2021 (start of the experiment) 17.0 ± 0.4 °C, summer 2021 24.0 ± 2.5 °C, autumn 2021 17.2 ± 1.8 °C, winter 2021–2022 9.6 ± 1.0 °C, spring 2022 15.2 ± 1.6 °C, and summer 2022 25.7 ± 2.7 °C. Corresponding cumulative precipitation values for these periods were 32, 23, 179, 21, 138, and 92 mm, respectively. All meteorological information was obtained from the Servei Meteorològic de Catalunya (https://meteo.cat/, accessed on 23 May 2024). Baseline soil properties were assessed before the experiment, showing a soil organic carbon content of 2.15%, total nitrogen of 0.19%, and a C/N ratio of 11.2.

The tomatoes analyzed in this study belong to two major varietal groups traditionally cultivated in Catalunya: Pebroter and Rosa. The Pebroter varieties Cornabel, Cuban Pepper, and Corno Andino are characterized by long, heavy, fleshy, horn-shaped fruits with a slightly sweet taste. In contrast, Roli Rosa, a representative of the Rosa variety, produces large, pink fruits highly appreciated for their distinctive flavor and texture, with sweetness prevailing over acidity. The Rosa variety has widespread market acceptance due to its superior quality [29].

### 2.3. Experimental Design

The experiment was conducted to evaluate the effects of different organically managed agronomic systems on tomato cultivation. In May 2021, four soil treatments were applied to the four tomato varieties [Cornabel (V1), Cuban Pepper (V2), Corno Andino (V3), and Roli Rosa (V4)], with four replicates per treatment. In treatment 1 (T1), 1.28 kg/m^2^ of woody residue compost (C/N ratio = 13) was incorporated into the soil. Treatment 2 (T2) served as the control, with no fertilizer applied. Treatments 3 and 4 (T3 and T4) involved the application of ramial chipped wood (C/N ratio = 40.5) at rates of 15 kg/m^2^ and 7.5 kg/m^2^, respectively. The RCW material was sourced from carbon-rich pruning residues collected in the municipality of Sant Boi de Llobregat.

All amendments were incorporated into the top 20 cm of soil using a rotary tiller (rotovator). All plots were rotary cultivated at the time of application. Treatments were randomly assigned to 16 plots, each measuring 1.5 × 7.5 m, separated by 1 m, and arranged along two 2 m-wide pathways to minimize positional or microclimatic effects. The soil surface was covered with biodegradable plastic, and a drip irrigation system was installed, providing the same water amounts to all plots to ensure uniform soil moisture across treatments.

In May 2022, prior to tomato planting, compost was reapplied to the T1 plots. Additionally, 210 kg of a commercial nitrogen-rich organic fertilizer (C/N ratio = 4) was added to the T2 plots, and 120 kg of the same fertilizer was applied to both T3 and T4. At that time, T1 and T2 plots were tilled once using the rotary cultivator, following common organic farming practices. In contrast, T3 and T4 plots were left untilled, based on the assumption that microbial mobilization of soil nitrogen would compensate for the lack of mechanical incorporation. Each of the 16 plots contained three plants per variety, spaced 45 cm apart.

Between the initial treatments and the enrichment of soil with nitrogen, different crops were cultivated. Sweet potatoes (*Ipomoea batatas* cv Beauregard) were grown from May to October 2021, followed by a combination of spinach (*Spinacia oleracea* L.) and fava beans (*Vicia faba* L) from October 2021 to May 2022. These crops were used as a complementary strategy for soil regeneration, aimed at improving tomato production and quality. The complete crop rotation and experiment management are detailed in Table 1.

This experiment was designed to compare the use of RCW as an organic amendment under no-tillage management with two conventional fertilization practices: plant-based compost (characterized by higher nutrient availability due to pre-decomposition) and organic pellet N fertilizer (characterized by slow-release N content). Both conventional systems (T1 and T2) were applied under tillage. This approach enables evaluation of how these different organic management strategies affect tomato yield and antioxidant-related phytochemical composition.

### 2.4. Tomato Sampling

During the August 2022 harvest, tomato samples were collected from the three plants of each variety within each treatment replicate. Fruits from the same variety and plot were pooled to obtain one composite sample per treatment-variety combination (*n* = 4 biological replicates). The samples were immediately transferred from the farm and frozen at −80 °C. Subsequently, samples from each treatment replicate were thawed, homogenized using a hand blender and lyophilized overnight (Telstar, Cryodos 50–2009, Terrassa, Spain). The lyophilized samples were stored in a freezer, then milled with a ball mill (15 s at 30 Hz; MM 500, Retsch, Haan, Germany) and stored again at −80 °C. A pooled quality control sample was prepared by combining material from a representative selection of all study samples.

### 2.5. Methanolic Extraction

Extraction was carried out according to Beer et al. (2024) [30], with some modifications. Briefly, 50 ± 0.5 mg of each sample was weighed in 2 mL tubes. Then, 500 μL of ice-cold methanol (MeOH) was added, followed by 20 µL of an internal standard mix (pool of eighteen compounds), whose details and corresponding chemical classes are provided in Table S1. The mixture was homogenized using a vortex for 5 s, then incubated in a thermal shaker (Ditabis HLC (Pforzheim, Germany), with 2 mL tube attachments) at 35 °C and 1400 rpm for 15 min. After incubation, samples were immediately placed on ice and centrifuged at 4 °C and 16,100 g for 5 min. Supernatants were filtered using a polytetrafluoroethylene (PTFE) syringe filter (0.2 µm, 4 mm). After vortexing for 5 s to ensure homogenization, 80 µL of the filtrate was transferred into an HPLC amber vial.

### 2.6. Metabolomic Profiling by High-Resolution Mass Spectrometry (UHPLC-QToF-MS)

Metabolomic analysis was performed using a 1290 Infinity LC system (Agilent Technologies, Waldbronn, Germany) coupled with a Triple TOF 5600 mass spectrometer (AB Sciex, Darmstadt, Germany). Separation was carried out on a Waters Acquity UPLC HSS T3 Premier column (2.1 × 150 mm; 1.8 µm) equipped with a VanGuard Acquity HSS T3 Premier pre-column (2.1 × 5 mm; 1.8 µm). Aqueous ammonium formate buffer (2 mM with 0.05 vol% formic acid) was used as eluent A and pure acetonitrile as eluent B at a total flow rate of 0.4 mL/min. The elution gradient was programmed as follows: 0.0–1.0 min, isocratic with 3% B; 1.0–14.0 min from 3 to 99% B; 14.0–20.0 min, isocratic with 99% B; 20.0–20.5 min, from 95 to 3% B; 20.5–25.5, isocratic with 3% B. The column oven was set at 40 °C and the injection volume was 2 μL. Mass spectrometry (MS) was performed in both positive and negative electrospray ionization (ESI) modes, selecting the following ionization source parameters: curtain gas 45 psi; ion spray voltage 5500 V and −4500 V; ion source gas-1 and gas-2 60 psi; and ion source gas-2 temperature 550 °C. The declustering potential was set to 70 V and −70 V, respectively. MS full scans were recorded over an *m/z* range of 100–1500 with an accumulation time of 150 ms and collision energies of 10 V and −10 V, respectively. MS/MS spectra were recorded in high sensitivity mode over an *m/z* range of 50–1500 with an accumulation time of 25 ms, a collision energy voltage of 35 V and −35 V, respectively, and a collision energy spread of 15 V.

All samples were freshly prepared on the respective measurement day. After the blanks and EQC samples were injected, the study samples were analyzed in randomized order in blocks of eight, each consisting of one PostCal sample, two QC samples, and five study samples, followed by an automatic mass calibration. The internal standard mix was injected into the solvent at the beginning of the sequence (before the first EQC sample) and at the end (after the last QC sample) to evaluate instrument performance. The analyses were performed using UPLC-QToF-MS under data-dependent acquisition (DDA) mode, first in positive and subsequently in negative ionization polarity. According to the COSMOS Metabolomics Standards Initiative [31], compounds identified by untargeted QTOF analysis reached level 2 annotation (putatively annotated compounds).

### 2.7. Post-Acquisition Data Analysis

Raw data files (.wiff and .wiff.scan) were converted into ABF format using the Reifycs ABF Converter and subsequently processed with MS-DIAL software (version 4.9) [32]. Automatic peak detection and compound annotation were carried out through spectral matching against a database developed at the Max Rubner-Institut. The mass range 80–1500 *m/z* was searched for peaks, with a minimum peak height threshold of 1000 cps in both ESI+ and ESI− modes. For peak centroiding, mass tolerances were set at 0.01 for MS and 0.05 Da for MS/MS. Retention time was included in the calculation of the total identification score. Mass accuracy tolerance for identification was set to 0.015 Da. Identification was based on accurate mass, ion species, and retention time, and a total identification score was calculated in MS-DIAL. A score threshold of 70% was applied, considering common ion adducts relevant to metabolomics. Gap filling was performed using the peak finder algorithm, with a 5 ppm tolerance for *m/z* values to recover missing peaks. To minimize false-positive identifications, all features detected in blank samples were excluded from the dataset prior to annotation. Additionally, only compounds with an identification score ≥ 90% were retained for further analysis, following COSMOS level 2 (putative) identification criteria.

### 2.8. Statistics

One-way ANOVA followed by post hoc analysis for tomato yields was performed using RStudio (version: 2025.05.0+496). Multivariate statistical analyses were conducted using MetaboAnalyst 6.0 (https://www.metaboanalyst.ca, accessed on 24 October 2025) [33]. Unsupervised principal component analysis (PCA) was used to evaluate variation between tomato varieties in all treatments. Variable Importance in Projection (VIP) scores were used to identify marker compounds. Fold change (FC) analysis of VIP markers (ANOVA, *p* < 0.05) was performed for each Pebroter tomato variety (V1, V2, V3) compared to V4, retaining significant compounds with a |Log_2_FC value| > ±2. In addition, Student’s *t*-tests and supervised orthogonal projections to latent structures discriminant analysis (OPLS-DA) were conducted using two classification criteria (T1 and T3), applied separately to each variety. VIP scores greater than 2 were considered indicative of marker compounds. Further FC analysis (Log_2_FC) of these VIP markers (*t*-test, *p* < 0.05) was performed for treatments T1 and T3 to identify significant marker compounds.

## 3. Results and Discussion

### 3.1. Tomato Yield

**Figure 1:**
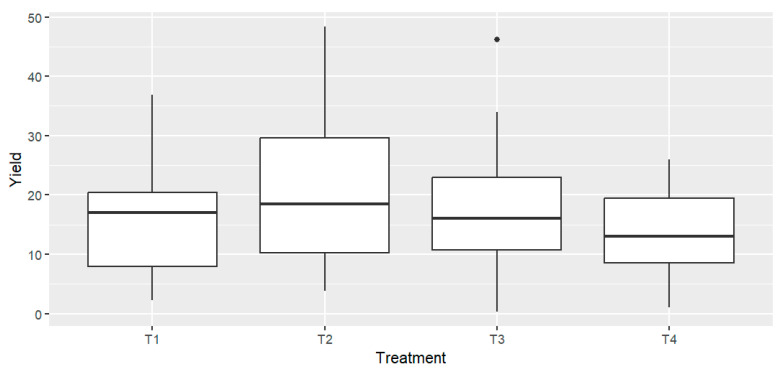
Tomato yields expressed as box plots for the different treatments. *n* = 4 biological replicates per treatment-variety.

At harvest, the total tomato yield from each treatment was collected and weighed. The average yield from four replicates per treatment was calculated and expressed in tons per hectare (Tn/ha) (Table 2). One-way ANOVA indicated no statistically significant differences in production among the different treatments across all tomato varieties F = 1.23, *p* = 0.307 (Figure 1). Assumptions of normality and homogeneity of variances were satisfied, as confirmed by Levene’s test (F = 1.03, *p* = 0.384). A two-way ANOVA was performed to evaluate the effects of fertilization treatment and tomato variety on yield and to assess potential treatment × variety interactions. Results showed no significant effect of treatment on yield (*p* = 0.052), while a significant effect of variety was observed (*p* < 0.001). The interaction between treatment and variety was not significant (*p* = 0.257), indicating that yield differences among treatments were consistent across varieties. Although numerical differences were observed between some treatments (e.g., Cornabel T2 = 32.65 Tn/ha vs. T4 = 16.44 Tn/ha), the overall variation was not statistically significant (Figure 1). However, the average yields revealed a consistent, though moderate, decrease under both high and low doses of RCW compared to the T2. This trend was observed across all four varieties (Table 2).

These findings are consistent with previous research by Soumare et al. (2002), who reported a decline in tomato growth and yield in the first cropping season after RCW application [25]. The reduction was attributed to nitrogen immobilization in the soil, caused by the high C/N ratio of RCW. In the present study, the applied RCW had a C/N ratio of 40.5, which likely induced similar immobilization effects. Notably, Soumare et al. (2002) also observed yield improvements in subsequent cropping, attributed to increased nitrogen availability as the RCW continued to decompose [25].

### 3.2. Phytochemical Variations Among the Tomato Varieties

Data obtained from UHPLC-QTOF-MS analysis were used for compound annotation with the Max Rubner-Institut in-house library, enabling putative (level 2, COSMOS) identification of metabolites. A total of 32,691 and 21,228 features were detected in positive and negative ionization modes, respectively. Features with identification scores ≥70% were initially retained (242 in positive and 221 in negative mode), while only those with scores ≥90% were included for final interpretation (93 in positive and 70 in negative mode). Background features detected in blanks were removed prior to analysis to minimize false discoveries. Overall, this approach enabled the putative identification of 163 compounds in tomatoes (93 in positive mode and 70 in negative mode). These were predominantly organic and phenolic acids (45), flavonoids (32), amino acids (31), alkaloids and amines (25), carbohydrates (20), fatty acids (4) and six other compounds. A full list of putatively identified compounds is available in the Supplementary Material (Table S2), including retention time, composite mass spectra, total identification score, and peak areas. Only compounds with a total score above 90% (based on retention time and accurate mass) were retained.

**Figure 2:**
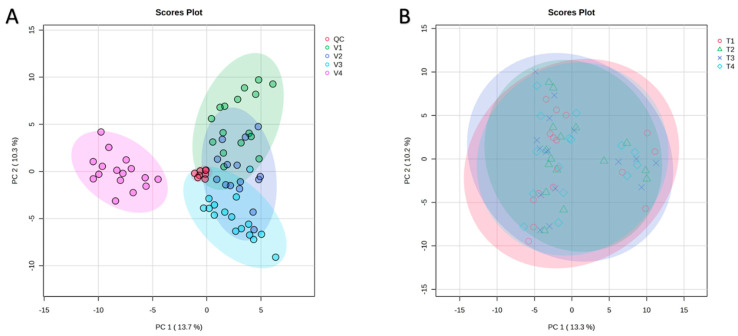
Principal component analysis (PCA) of (**A**)—The four varieties (V1: Cornabel, V2: Cuban Pepper, V3: Corno Andino, and V4: Roli Rosa) showing the clear separation of V4 from the Pebroter group; and (**B**)—The four fertilization treatments in all varieties, showing the four treatments clustered together. *n* = 4 biological replicates per treatment-variety.

PCA was performed on all samples to assess variation and correlations among the four tomato varieties (Figure 2A). The first two principal components explained 13.7% (PC1) and 10.3% (PC2) of the total variance. In the PCA score plot, V1, V2, and V3 (all belonging to the Pebroter group) formed a tight cluster, whereas V4 (Rosa group) was clearly separated along PC1, reflecting distinct metabolomic profiles between the two varietal groups. The QC samples clustered closely at the center of the PCA plot, demonstrating excellent analytical stability and reproducibility of the UHPLC-QToF-MS measurements. The PERMANOVA test supported these findings, revealing significant differences among varieties (F = 67.506, R^2^ = 0.8036, *p* = 0.001, based on 999 permutations).

To identify key discriminatory variables among the four tomato varieties, a one-way ANOVA followed by post hoc tests was applied to all identified compounds and their corresponding peak intensities (areas). The analysis revealed significant differences (*p* < 0.05) among the varieties. Of the 163 identified compounds, 115 showed statistically significant differences between at least two varieties. To ensure statistical robustness, both raw and FDR-corrected p-values were calculated, and interpretation was based on the adjusted results. A complete list of these compounds, along with *p*-values, false discovery rates, and Fisher’s least significant difference comparisons, is provided in the Supplementary Material (Table S3).

To identify discriminant metabolites, fold change analysis was conducted on the significant features (ANOVA, *p* < 0.05), comparing each Pebroter variety (V1, V2, V3) to Roli Rosa (V4). Compounds with a |Log_2_FC value| > +2 were considered significantly different. The results (Table 3) indicate that 37 compounds showed significant variation, primarily flavonoids (25) and phenolic acids (5). These findings are consistent with previous research [4], which identified flavonoids and phenolic acids as potential chemotaxonomic markers to differentiate tomato varieties. Additionally, six alkaloids and amines differed significantly between varieties, whereas no amino acids showed significant variation. This contrasts with a previous report that proposed specific amino acids as biochemical markers to distinguish between 12 Italian tomato varieties [34]. Another study suggested that isotopic analysis of amino acids may serve as a novel tool for authenticating organic tomatoes [35].

Among the 37 discriminant compounds, 30 exhibited higher intensities in the Pebroter varieties than in Roli Rosa (Log_2_FC ≥ +2). In contrast, only seven compounds (orientin, phloretin glucoside, kaempferol, quercetin dihydrate, cyanidin, quercetin diglucoside, and cucurbitacin D) were more abundant in Roli Rosa (Log_2_FC ≤ −2). Although some amines and alkaloids also differed between varieties, their Log_2_FC values only ranged from 2 to 3. Notably, seven flavonoids showed substantial variation, with Log_2_FC values ≥ 4, indicating more than a fourfold increase in intensity in the Pebroter varieties: eriodictyol, naringenin glucoside, naringenin chalcone, orobol (isoluteolin), eriodictyol glucoside, phloretin, and apigenin. Their structural similarity (Figure S1) suggests enhanced activity of flavonoid biosynthetic enzymes in Pebroter.

**Figure 3:**
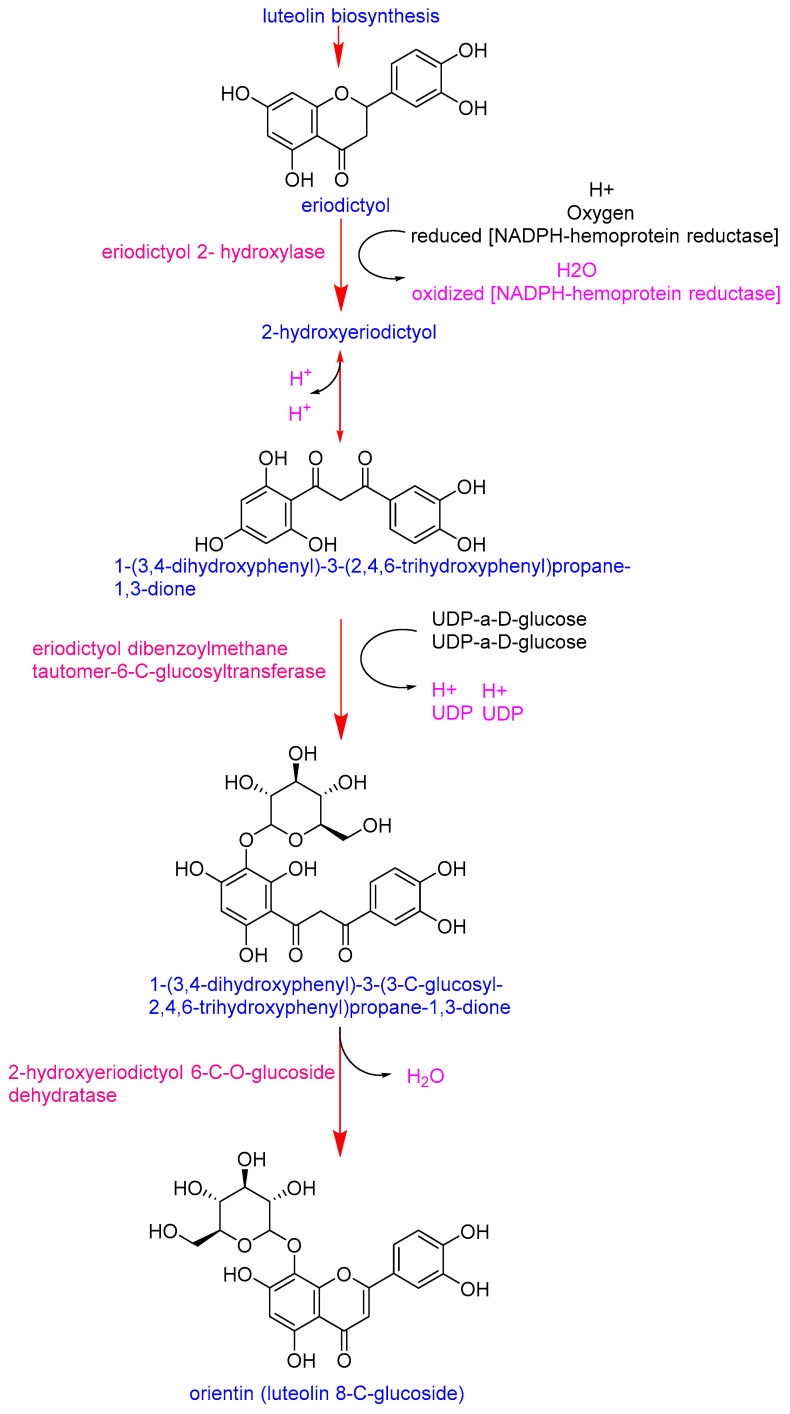
Biosynthetic pathway of orientin [36].

Eriodictyol emerged as the most distinctive marker, with significantly higher intensities in the Pebroter varieties (Log_2_FC > 5). In contrast, luteolin-8-C-glucoside (orientin) was more abundant in Roli Rosa (Log_2_FC < −2). Examination of the orientin biosynthetic pathway (Figure 3) shows that its synthesis in plants begins with eriodictyol and involves two key enzymes: eriodictyol 2-hydroxylase and eriodictyol dibenzoylmethane tautomer-6-C-glucosyltransferase [36]. The accumulation of eriodictyol in Pebroter varieties, without conversion to orientin (as observed in Roli Rosa), suggests a deficiency or low expression of the genes encoding these enzymes in Pebroter. Although this metabolic interpretation is consistent with known biosynthetic routes, experimental validation through targeted gene expression or enzyme activity assays would be required to confirm these observations. Quantifying the activity of these two enzymes could therefore provide a useful biochemical marker to differentiate between the two varietal groups. These results are consistent with previous studies in which enzyme profiling effectively distinguished between tomato varieties, reflecting their genetic and metabolic diversity [37].

Furthermore, five phenolic compounds (homovanillic acid, hydroxyphenylacetic acid, hydroxytyrosol, neochlorogenic acid, and chlorogenic acid; Figure S2) showed significant variation between Pebroter and Roli Rosa varieties. Their elevated intensities in Pebroter suggest increased activity or abundance of the enzymes involved in their biosynthetic pathways. Among them, homovanillic acid (Log_2_FC ≈ 5.2) and hydroxyphenylacetic acid (Log_2_FC > 4) exhibited the most pronounced differences, with homovanillic acid presenting the largest fold change among phenolic acids.

**Figure 4:**
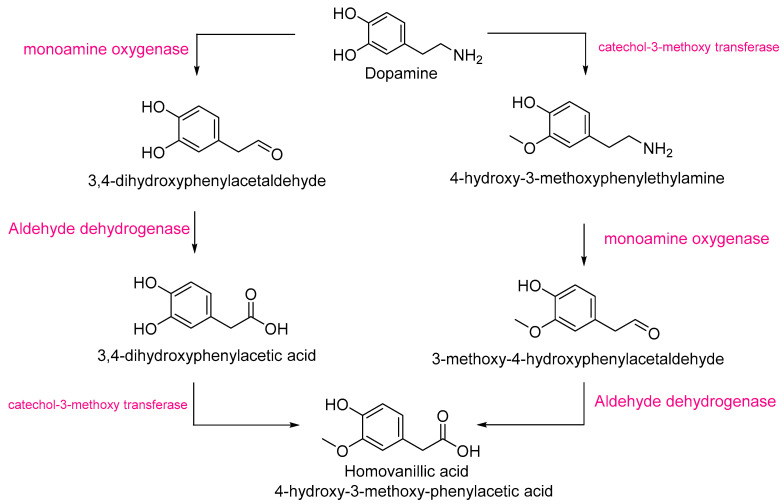
The homovanillic acid biosynthetic pathway starts from dopamine [38].

Homovanillic acid is a metabolite of dopamine, which was also significantly more abundant in Pebroter (Log_2_FC ≈ +2). The biosynthesis of homovanillic acid proceeds via dopamine degradation [38] and involves three enzymes: monoamine oxidase (MAO), catechol-*O*-methyl transferase (COMT), and aldehyde dehydrogenase (Figure 4). Both MAO [39,40] and COMT [41] have been previously identified in tomato. The higher intensities of homovanillic acid and dopamine in Pebroter could imply enhanced activity or expression of MAO, COMT, and/or aldehyde dehydrogenase compared to Roli Rosa.

### 3.3. Effects of Fertilization Treatments on the Phytochemical Composition of Tomato Varieties

In contrast to the pronounced varietal differences, PCA revealed no significant separation among the four fertilization treatments across all tomato varieties, with treatments clustering closely together and exhibiting similar distribution patterns (Figure 2B). This observation was supported by one-way ANOVA of the 163 identified compounds, which showed no significant differences in peak intensities (areas) between treatments.

To investigate treatment effects in more detail, we analyzed each variety individually. A *t*-test comparing the two organic systems with different doses of RCW (T3 vs. T4) revealed no significant differences in compound abundances or peak areas in any variety, indicating that increasing the RCW concentration beyond 7.5 kg/m^2^ does not influence the phytochemical composition of the tomato fruits. To further examine the effect of using woody residues as an organic fertilizer, OPLS-DA was performed separately for T3 vs. T1 (compost fertilizer with a C/N ratio of 15) and T3 vs. T2.

However, empirical validation using 100 permutation tests indicated that the predictive ability of the OPLS-DA models was not statistically significant for any variety: V1 Q^2^ *p* = 0.86, R^2^Y *p* = 0.91; V2 Q^2^ *p* = 0.93, R^2^Y *p* = 0.18; V3 Q^2^ *p* = 0.84, R^2^Y *p* = 0.54; V4 Q^2^ *p* = 0.41, R^2^Y *p* = 0.44. The lack of statistically significant Q^2^ and R^2^Y values across all varieties indicates that the supervised OPLS-DA models are not overfitted, and therefore, the identified treatment-associated metabolites should be interpreted as preliminary observations. Consequently, while per-variety OPLS-DA identified metabolites that respond to fertilization, these findings are considered exploratory and require confirmation with larger sample sizes.

#### 3.3.1. Effect of Using Ramial Chipped Wood (T3) Versus Commercial Woody Residue Compost (T1) on Tomato Varieties

**Figure 5:**
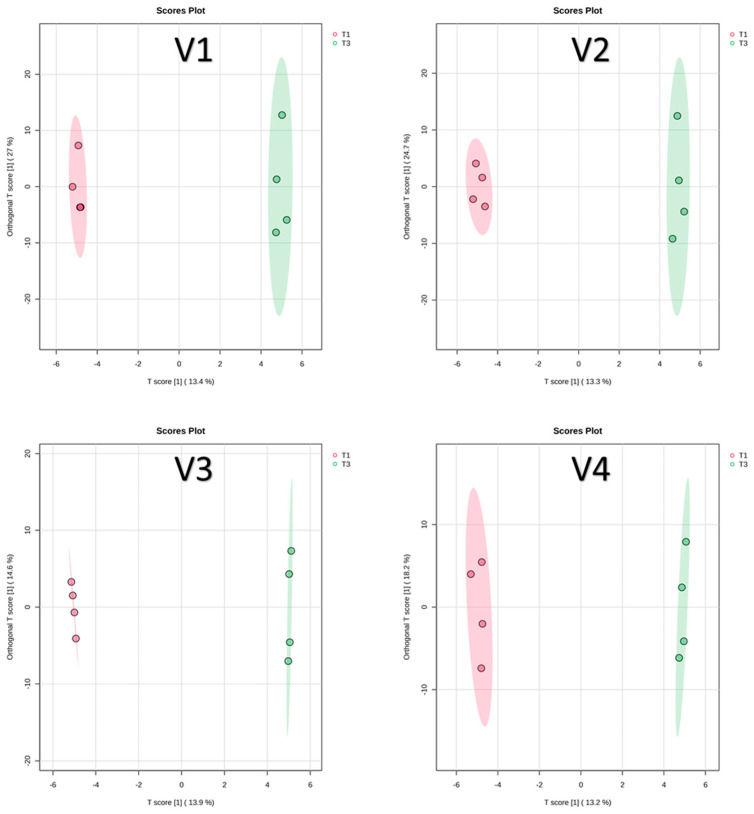
OPLS-DA score plots for T1 versus T3. V1: Cornabel, V2: Cuban Pepper, V3: Corno Andino, and V4: Roli Rosa. T1: compost fertilizer from woody residues. T3: Ramial chipped wood (15 kg/m^2^). *n* = 4 biological replicates per treatment-variety.

Low fertilization levels are known to affect both tomato yield and phytochemical composition [42]. In this study, a supervised OPLS-DA was conducted to identify key compounds that differentiate between compost (T1) and RCW (T3) treatments within each of the four varieties. The OPLS-DA score plots for T1 versus T3 in all varieties are shown in Figure 5.

In the Cornabel variety (V1), VIP analysis identified 10 marker compounds that significantly distinguished between T1 and T3 (Figure S3). Additionally, Log_2_FC values were calculated to quantify the differences in marker abundance between treatments (Table 4). Among these, *p*-coumaric acid showed the highest VIP score (>2.4), indicating greater abundance under the RCW treatment (T3) compared to compost (T1). This likely reflects the lower nitrogen availability in T3 soils and is consistent with previous findings in Cornabel, where replacing nitrogen-rich with nitrogen-poor fertilizers increased *p*-coumaric acid levels from 0.48 to 0.94 mg/kg [9]. While soil mineral N was not directly measured in this study, the higher *p*-coumaric acid in T3 may reflect reduced nitrogen availability and stress-induced phenolic synthesis, a common plant defense response [43].

Similarly, abscisic acid (an organic acid) and trigonelline (the only alkaloid marker identified in Cornabel) showed higher intensities in T3 than in T1. Carbohydrates such as kestose and glucovanillin were also more abundant under RCW treatment, each showing approximately a onefold increase relative to T1. This finding contrasts with previous studies reporting that soluble carbohydrate levels are not affected by increasing the C/N ratio [44].

The Cuban Pepper variety responded differently to RCW treatment compared to Cornabel. Flavonoid intensities decreased, with quercetin diglucoside and luteolin-8-glucoside (orientin) showing significant reductions (Log_2_FC values of 1.5 and 1.9, respectively). This decline in flavonoid glycosides is consistent with previous reports of decreased rutin and naringenin glucoside in Cuban Pepper under low-nitrogen organic fertilization [9]. Similarly, carbohydrates such as sucrose and sorbitol derivatives also declined under the woody residue treatment (T3). In contrast, the isopentylamine content in T3 was approximately double that of T1. The fatty acid nonanoic acid increased under T3 in Cuban Pepper but decreased in Cornabel. These differing metabolic responses to the same treatment likely reflect genetic differences between the varieties.

The Corno Andino variety responded similarly to Cornabel under T3, though with distinct increases in the flavonoid luteolin and the organic acid succinic acid. In Roli Rosa, six marker compounds had significant VIP scores, predominantly organic acids (pyruvic acid, dihydroxybenzoic acid, homovanillic acid, and adipic acid), which increased by approximately onefold under the compost treatment (T1).

Across all varieties, the high-dose woody residue treatment (T3) consistently increased the abundance of organic acids and amines (Figure S4), whereas flavonoid and carbohydrate responses were more variety-specific. These findings suggest that flavonoid accumulation is more strongly influenced by the tomato variety, whereas organic acids and amines appear more responsive to nutritional stress induced by the application of woody by-products.

#### 3.3.2. Effect of Woody Residue Versus Commercial Nitrogen-Rich Pellets on Tomato Fruit Composition

Scharenbroch & Watson (2014) demonstrated that RCW is an effective, cost-efficient organic amendment for improving soil quality and stimulating tree growth [45]. In this study, we examined whether the application of woody by-products (T3 and T4) influenced the chemical composition of tomato fruits compared to a conventional nitrogen-rich fertilizer (T2). OPLS-DA revealed a clear metabolic separation between T2 and T3 across all four tomato varieties (Figure S5). Potential marker compounds, identified based on VIP(t) scores (>2), are listed in Table 5 and Figure S6 with the corresponding Log_2_FC values.

In the Cornabel variety (V1), treatment with low-nitrogen RCW (T3) led to reduced intensities of several phenolic compounds and organic acids. Specifically, dihydroxyphenyl lactic acid and isoferulic acid decreased by approximately half-fold. Succinic acid, lactic acid and the amino acid gamma-aminobutyric acid (GABA) were also reduced under T3 compared to the nitrogen-rich treatment (T2). In contrast, flavonoids such as iso-schaftosid and eriodictyol glucoside were more abundant under T3.

In the Cuban Pepper variety, a decrease in nitrogen-containing compounds such as malonyl tryptophan and feruloyl tyramine was observed, with an approximate onefold reduction (Log_2_FC around 0.9) under the high C/N RCW treatment.

In the Corno Andino variety, nine marker compounds showed significant variation between treatments (*p*-value < 0.05). Among these, two flavonoids (taxifolin and quercetin dihydrate) displayed a notable increase under the low-nitrogen RCW treatment (Log_2_FC values of −1.7, and −1.5, respectively). In contrast, most nitrogen-containing compounds, including amines and amino acids, decreased in intensity, except for L-homoglutamic acid, which showed a modest half-fold increase.

The Roli Rosa variety showed a similar pattern to Pebroter group, with reduced intensities of phenolic acids (e.g., caffeic acid) and amines (e.g., *p*-coumaroyl tyramine) (Log_2_FC values of 0.74 and 1.49, respectively). Notably, L-glutamic acid increased, mirroring the trend observed for L-homoglutamic acid in Corno Andino. These two compounds were the only amino acids that increased in response to wood chip fertilization. As observed for the T1–T3 comparison, permutation testing for the T2–T3 OPLS-DA models showed no statistically significant predictive ability (V1–V4 Q^2^ *p* = 0.19–0.92; R^2^Y *p* = 0.11–1.00). These findings suggest that the models are not overfitted and that the detected treatment-related metabolic variations should be interpreted as exploratory, requiring confirmation with larger sample sizes.

Overall, consistent with previous findings [46], the RCW treatment tended generally to increased flavonoid levels across all cultivars while decreasing phenolic acids, organic acids, and most nitrogen-containing compounds, except for glutamic acid and its isomer, homoglutamic acid, compared to the unfertilized control.

Finally, post-harvest stability parameters, including physiological loss in weight (PLW), were not evaluated in this study, as all tomato samples were harvested at full maturity, immediately frozen, and analyzed to preserve their native metabolite composition. Consequently, no conclusions can be drawn regarding post-harvest performance or storage behavior under RCW fertilization. Nevertheless, since RCW application may influence the antioxidant profile of tomatoes, future studies should explore its potential effects on post-harvest stability. Moreover, the RCW and compost materials used originated solely from clean plant residues collected from city gardens and forests, minimizing the risk of *E. coli* or other microbial contamination.

## 4. Conclusions

In this study, we analyzed four tomato varieties—Cornabel, Cuban Pepper, Corno Andino, and Roli Rosa—under contrasting organic farming practices using untargeted metabolomic profiling via UHPLC-QToF-MS and statistical analyses, with a particular focus on antioxidant-related metabolites. 163 compounds were putatively identified via spectral matching based on accurate mass, ion species, and retention time, using a reference database from the Max Rubner-Institut. Among them, 37 compounds were identified as biomarkers capable of distinguishing between the Pebroter and Roli Rosa varieties: 30 polyphenolic compounds were more abundant in Pebroter, while seven phenolic compounds and amines were enriched in Roli Rosa. Notably, eriodictyol was highly abundant in Pebroter, whereas Roli Rosa accumulated orientin, suggesting lower expression or activity of enzymes responsible for converting eriodictyol to orientin in Pebroter, while further validation through targeted gene expression or enzyme activity analyses is recommended.

Although PCA did not reveal statistically significant differences across fertilization treatments, variety-specific responses were observed. RCW showed tendency for increasing the abundance of several antioxidant compounds and related metabolites such as phenolic and organic acids (e.g., *p*-coumaric acid, abscisic acid, succinic acid, pyruvic acid, and dihydroxybenzoic acid), compared to commercial compost; however the concentration of RCW had no significant effect on the overall phytochemical composition. RCW also slightly increased levels of nitrogen-containing compounds, such as amines and alkaloids, relative to the compost. Compared to the control, flavonoid levels generally tended to increase under RCW, whereas phenolic acids tended to decline, though these effects were variety-dependent. Importantly, first-season yield reductions, variability among varieties, differences in nitrogen availability, and the use of tillage in T1/T2 versus no tillage in T3/T4 highlight the need for multi-season trials and further studies to confirm the agronomic performance of RCW under diverse management conditions. Moreover, the multivariate models comparing fertilization treatments indicated no separation, suggesting that further investigation is needed to confirm these preliminary metabolic trends. Overall, our findings support the use of RCW as a sustainable organic fertilizer that may influence the antioxidant metabolite profile of tomatoes; further studies with unfertilized controls are needed.

## Figures and Tables

**Table 1:** Crop rotation and management carried out in the experiments.

	T1	T2	T3	T4
May 2021	12.8 Tn/ha of compost of woody plant residues rich in nitrogen (C/N ratio 15.3) (168 kg N/ha).	Control, no fertilizers	15 kg/m^2^ of woody plant residues.	7.5 kg/m^2^ of woody plant residues.
May to October 2021	Sweet potato (*Ipomoea batatas* var *Beauregard*).			
October 2021 to April 2022	Spinach (*Spinacia oleracea*) and fava beans (*Vicia faba*).			
May 2022	Another application of compost.	210 kg of nitrogen-rich organic fertilizer (C/N ratio 4).	120 kg of nitrogen-rich fertilizer.	120 kg of nitrogen-rich fertilizer.
May to August 2022	Tomato (*Solanum lycopersicum* var Cornabel, Cuban Pepper, Corno andino and Roli Rosa).			

**Table 2:** Average tomato fruit yield for each treatment for the four investigated tomato varieties expressed in Tn/ha.

Tomato Variety	Treatment	Average Tomato Yield ± SD	* *n*
Cornabel	T1	26.02 ± 7.62	4
	T2	32.65 ± 11.05	4
	T3	28.30 ± 12.75	4
	T4	16.44 ± 4.13	4
Cuban Pepper	T1	7.00 ± 1.85	4
	T2	9.46 ± 3.98	4
	T3	4.32 ± 3.15	4
	T4	5.82 ± 3.43	4
Corno Andino	T1	13.60 ± 7.30	4
	T2	13.15 ± 7.36	4
	T3	14.03 ± 3.35	4
	T4	10.57 ± 3.12	4
Roli Rosa	T1	13.71 ± 7.73	4
	T2	24.22 ± 7.17	4
	T3	22.75 ± 7.94	4
	T4	20.88 ± 4.58	4

* *n*. is the number of biological replicates.

**Table 3:** Marker compounds from each chemical class with ANOVA *p* < 0.05. Fold change and Log_2_FC comparing each Pebroter variety (V1, V2, V3) with the Roli Rosa variety (V4). The compounds were putatively identified based on accurate mass and retention time.

		*p* Value	FDR	V1/V4		V2/V4		V3/V4	
	Class			Fold Change	log2(FC)	Fold Change	log2(FC)	Fold Change	log2(FC)
Eriodictyol	Flavonoids	2.01 × 10^−11^	3.23 × 10^−10^	44.676	5.4814	57.623	5.8486	21.937	4.4553
Naringenin glucoside	Flavonoids	6.90 × 10^−7^	3.52 × 10^−6^	26.48	4.7268	8.3228	3.0571	3.4295	1.778
Naringenin chalcone	Flavonoids	7.59 × 10^−15^	2.64 × 10^−13^	26.099	4.7059	46.675	5.5446	24.969	4.6421
Orobol (Isoluteolin)	Flavonoids	6.97 × 10^−8^	5.20 × 10^−7^	24.323	4.6042	21.557	4.4301	13.354	3.7392
Eriodictyol glucoside	Flavonoids	0.0004988	0.0012411	19.373	4.276	2.1239	1.0867		
Epigallocatechin	Flavonoids	4.33 × 10^−6^	1.56 × 10^−5^	11.28	3.4957	3.0995	1.632	2.5137	1.3298
Phloretin	Flavonoids	3.28 × 10^−5^	0.00010231	9.9355	3.3126	31.276	4.967	16.903	4.0792
Isorhamnetin rutinoside	Flavonoids	9.21 × 10^−8^	6.64 × 10^−7^	7.38	2.8836	6.5918	2.7207	7.9735	2.9952
Naringenin	Flavonoids	1.70 × 10^−6^	7.58 × 10^−6^	7.2215	2.8523	11.48	3.5211	9.5422	3.2543
Tiliroside	Flavonoids	0.002343	0.0047085	7.0406	2.8157	4.5574	2.1882	8.8628	3.1478
Apigenin	Flavonoids	3.06 × 10^−12^	7.11 × 10^−11^	6.4713	2.6941	35.205	5.1377	27.615	4.7874
Quercetin-rutinoside-glucoside	Flavonoids	1.04 × 10^−16^	7.22 × 10^−15^	5.9906	2.5827	5.3633	2.4231		
Isoschaftoside	Flavonoids	6.58 × 10^−7^	3.51 × 10^−6^	5.8309	2.5437	6.3154	2.6589	3.0446	1.6062
Phloretin-xylosyl-glucoside	Flavonoids	7.90 × 10^−9^	7.86 × 10^−8^	4.4239	2.1453	4.7253	2.2404	5.07	2.342
Hispidulin	Flavonoids	1.69 × 10^−8^	1.47 × 10^−7^	4.0792	2.0283	5.1687	2.3698	9.5069	3.249
Luteolin-8-*O*-glucoside (orientin)	Flavonoids	4.32 × 10^−9^	4.52 × 10^−8^	0.18491	−2.4351	0.22792	−2.1334	0.24266	−2.043
Phloretin glucoside	Flavonoids	0.00020707	0.00054781	0.16024	−2.6417				
Kaempferol	Flavonoids	0.0024796	0.0049356	0.12376	−3.0144	0.18835	−2.4085		
Luteolin	Flavonoids	1.45 × 10^−6^	6.73 × 10^−6^	2.6764	1.4203	7.4521	2.8976	4.2352	2.0824
Quercetin xyloside	Flavonoids	0.00054223	0.0012829	2.9479	1.5597	4.0776	2.0277	2.4729	1.3062
Kaempferol glucoside	Flavonoids	0.008785	0.014927			4.0024	2.0009		
Quercetin (Dihydrate)	Flavonoids	4.03 × 10^−5^	0.00011698	0.27247	−1.8758	0.2467	−2.0192	2.4737	−1.3067
Cyanidin	Flavonoids	1.10 × 10^−9^	1.35 × 10^−8^	0.26903	−1.8941	0.22967	−2.1224	0.27402	−1.8677
Quercetin diglucoside	Flavonoids	1.18 × 10^−13^	3.53 × 10^−12^	0.27236	−1.8764	0.17517	−2.5132	0.22432	−2.1564
Isorhamnetin glucoside	Flavonoids	0.0008438	0.0018564	2.8579	1.515	5.3633	2.4231	6.013	2.5881
Kukoamine A	Amines	3.28 × 10^−5^	0.00010231	31.832	4.9924	11.078	3.4697	4.8105	2.2662
Serotonin	Amines	3.74 × 10^−12^	7.82 × 10^−11^	7.5597	2.9183	2.6199	1.3895	2.7762	1.4731
Dopamine	Amines	1.07 × 10^−7^	6.97 × 10^−7^	7.2701	2.862	3.2964	1.7209	2.6482	1.405
Feruloyl tyramine	Amines	2.07 × 10^−7^	3.39 × 10^−6^			4.0428	2.0153		
Hydroxy-methoxyphenylacetic acid (Homovanillic acid)	Phenols and organic acids	1.44 × 10^−8^	1.31 × 10^−7^	36.069	5.1727	34.674	5.1158	41.205	5.3647
Hydroxyphenylacetic acid	Phenols and organic acids	9.37 × 10^−7^	4.55 × 10^−6^	16.299	4.0268	19.242	4.2662	18.108	4.1786
Hydroxytyrosol	Phenols and organic acids	1.94 × 10^−6^	8.11 × 10^−6^	5.4923	2.4574				
Neochlorogenic acid	Phenols and organic acids	1.26 × 10^−7^	7.97 × 10^−7^	4.7016	2.2332			2.1873	1.1291
Chlorogenic acid (Hemihydrat)	Phenols and organic acids	0.00055043	0.0012829	4.679	2.2262	5.0156	2.3264		
Glucovanillin	Carbohydrates	1.37 × 10^−9^	1.59 × 10^−8^	9.6322	3.2679	8.9704	3.1652	6.0604	2.5994
Cucurbitacin D	Triterpenes	4.52 × 10^−15^	1.89 × 10^−13^	0.2066	−2.2751	0.46474	−1.1055	0.48138	−1.0548
Resveratrol	Stilbenoids	1.15 × 10^−15^	6.03 × 10^−14^	3.7393	1.9028	26.1	4.706	3.4232	1.7753

**Table 4:** OPLS-DA VIP scores, and Log_2_fold changes for T1 versus T3, along with the chemical class of each marker compound. The compounds were putatively identified based on accurate mass and retention time.

T1 Is the Compost and T3 Is Ramial Chipped Wood			
Compound	Category	* Log_2_FC	VIP[t] OPLS-DA
Variety 1 (Cornabel)			
*p*-Coumaric Acid	hydroxyl phenols	1.146529	2.407687
Nonanoic Acid	fatty acids	−0.9427	2.399184
Abscisic acid	organic acids	1.008865	2.381753
Kestose	sugar	0.154476	2.078115
Trigonelline	alkaloids	0.878093	2.055114
Pantothenic acid	vitamin B	0.417785	1.983722
Isoschaftoside	flavonoid glycoside	1.138413	1.984392
Pyrrolidinone	heterocyclic organic comp	−1.21666	1.956484
Erythronolactone	lactone	−0.43937	1.977656
Glucovanillin	glycosides	0.788786	1.965855
Variety 2 (Cuban Pepper)			
Quercetin diglucoside	flavonoids	−1.56143	2.130441
Nonanoic acid	fatty acids	0.53435	2.050532
D-Saccharose	sugar	−0.69369	2.060782
Isopentylamine	amines	1.037912	2.059114
Tomatin	carbohydrates	−1.25702	2.047888
Indol carbaldehyde	indole	1.326053	2.092906
Hydroxy-methoxyphenyl acetic acid	organic acids	−0.99027	1.969829
Anhydro sorbitol	sugar	−0.39654	2.010885
Luteolin-8-C-glucoside	flavonoids	−1.90158	1.923035
Variety 3 (Corno Andino)			
Succinic acid	organic acids	0.493492	2.460384
L-Asparagine	amino acids	−0.41132	2.265669
Luteolin	flavonoids	1.658417	2.19627
Variety 4 (Roli Rosa)			
Pyruvic acid	organic acids	1.234785	2.450444
Dihydroxybenzoic acid	organic acids	0.916453	2.272568
Apigenin	flavonoids	−2.2098	2.257424
Hydroxyproline betaine	amino acids	0.897212	2.155159
Hydroxy-methoxyphenylacetic acid	organic acids	0.92976	1.992463
Adipic acid	organic acids	0.971178	1.973279

* Positive Log_2_FC values indicate higher abundance in T3 compared to T1. Negative Log_2_FC values indicate lower abundance in T3 compared to T1.

**Table 5:** OPLS-DA VIP scores, and Log_2_fold changes for T2 versus T3, along with the chemical class of each marker compound. The compounds were putatively identified based on accurate mass and the retention time.

T2 Is the Control and T3 Is RCW			
Compound	Category	* Log_2_FC	VIP[t] OPLS-DA
Variety 1 (Cornabel)			
Gamma aminobutyric acid	amino acids	0.50032	2.358385
Iso-schaftoside	flavonoids	−1.2532	2.323798
Succinic acid	organic acids	0.454959	2.205357
Eriodictyol glucoside	flavonoids	−1.66415	2.222554
Dihydroxyphenyl lactic acid	hydroxyl phenols	0.756158	2.183703
Abscisic acid	organic acids	−0.67688	2.186506
Feruloyl glucoside	glycosides	1.086971	2.142704
Lactic acid	organic acids	0.940623	2.152517
Isoferulic acid	hydroxyl phenols	0.555847	2.054568
Variety 2 (Cuban Pepper)			
Malonyl-tryptophan	amino acids	0.909838	2.455417
Glucoheptonic acid lactone	lactones	1.049061	2.069926
Feruloyl tyramine	phenolic amines	0.956465	2.032768
Variety 3 (Corno Andino)			
Indol carbaldehyde	heterocyclic indoles	−0.93623	2.456818
Taxifolin	flavonoids	−1.79987	2.293026
Methyl succinic acid	organic acids	−0.31006	1.991324
Trigonelline	alkaloids	0.788205	1.990487
L-Tryptophan	amino acids	0.265948	1.996145
Tyramine	phenolic amines	0.880057	1.97969
L-Homoglutamic acid	amino acids	−0.43322	1.90232
Quercetin dihydrate	flavonoids	−1.51721	1.93636
L-lysine	amino acids	0.450673	1.898549
Variety 4 (Roli Rosa)			
Erythronolactone	lactones	0.547699	2.042465
Erythrodiol	triterpenes	−1.27068	1.859939
*p*-cumaroyl tyramin	phenolic amines	1.492106	1.834817
L-Glutamic acid	amino acid	−0.78378	1.85933
Caffeic acid	hydroxyl phenols	0.747991	1.777343

* Positive Log_2_FC values indicate higher abundance in T3 compared to T2. Negative Log_2_FC values indicate lower abundance in T3 compared to T2.

## Data Availability

Data is contained within the article and Supplementary Material.

## Supplementary Materials

The following supporting information can be downloaded at: https://www.mdpi.com/article/10.3390/antiox14111330/s1, Figure S1: Chemical structures of the main markers; Figure S2: Phenolic compounds showed significant variation between the Pebroter and Roli Rosa varieties; Figure S3: OPLS-DA VIP scores for the selected compounds in T1 versus T3; Figure S4: Box-plots of organic acids and amines show variations between T1 versus T3; Figure S5: OPLS-DA score plots for T2 versus T3. V1: Cornabel; Figure S6: OPLS-DA VIP score for the selected compounds in T2 versus T3; Table S1: Internal standards used in *UHPLC-QToF-MS* evaluation of instrument performance; Table S2: List of identified compounds; Table S3: Differentially accumulated metabolites between treatments with corresponding raw and FDR-adjusted p-values and Fisher’s LSD comparisons.
