# Supplementary material for: Sustainable Fertilization with Ramial Chipped Wood Enhances Antioxidant Profiles in Tomato Varieties: An Untargeted Metabolomics Approach
Source: Antioxidants (Basel). 2025 Nov 5;14(11):1330. doi: 10.3390/antiox14111330 (PMC12649442; doi:10.3390/antiox14111330)
Supplement: Supplementary file 1 [file antioxidants-14-01330-s001.zip › Supplementary material.pdf]

Sustainable Fertilization with Ramial Chipped Wood Enhances Antioxidant Profiles in Tomato Varieties: An Untargeted Metabolomics Approach

Mohamed M. Abuhabib [a,b], *Clara Abarca-Rivas* [a,b,c], *Julián Lozano-Castellón* [a,b,c], *Anna Vallverdú-Queralt* [a,b,c], *Johana González-Coria* [b,d], *Sebastian T. Soukup* [e], *Rosa M. Lamuela-Raventós* [a,b,c], *Maria Pérez* [a,b,c,]*, *and Joan Romanyà* [b,c,d,]*

[a] Polyphenol Research Group, Department of Nutrition, Food Science and Gastronomy, Faculty of Pharmacy and Food Sciences, University of Barcelona, 08028, Barcelona, Spain.

[b] Institute of Nutrition and Food Safety (INSA-UB), University of Barcelona, 08028, Barcelona, Spain

[c] CIBER Physiopathology of Obesity and Nutrition (CIBEROBN), Institute of Health Carlos III, 28029 Madrid, Spain.

[d] Department of Biology, Health and the Environment, Faculty of Pharmacy and Food Sciences, University of Barcelona, 08028, Barcelona, Spain.

[e] Department of Safety and Quality of Fruit and Vegetables, Max Rubner-Institut (MRI) – Federal Research Institute of Nutrition and Food, Department of Safety and Quality of Fruit and Vegetables, 76131 Karlsruhe, Germany.

* Corresponding authors:

Joan Romanyà
Tel: +34 93 402 44 94
Facultat de Farmàcia i Ciències de l'Alimentació
Universitat de Barcelona
Avgda. Joan XXIII 27-31
08028 Barcelona
E-mail: jromanya@ub.edu
https://orcid.org/0000-0002-4519-9653

Maria Pérez
Tel: +34 93 403 93 62
Facultat de Farmàcia i Ciències de l'Alimentació
Universitat de Barcelona
Campus de l'Alimentació de Torribera
Carrer de Prat de la Riba, 171
08921 Santa Coloma de Gramenet, Barcelona
E-mail: mariaperez@ub.edu
https://orcid.org/0000-0001-5775-6472

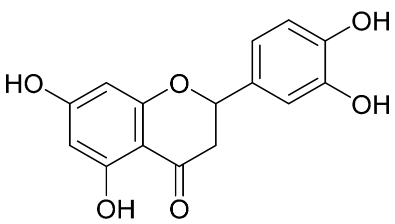

eriodictyol

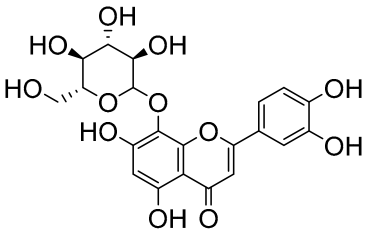

Orientin
luteolin 8-C-glucoside

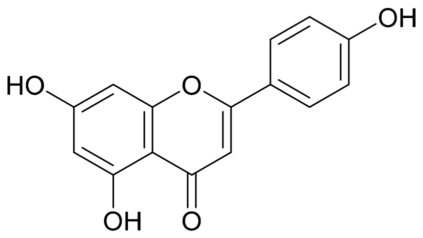

Apigenin

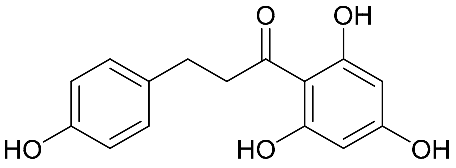

Phloretin

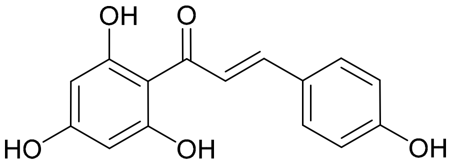

Naringenin chalcone

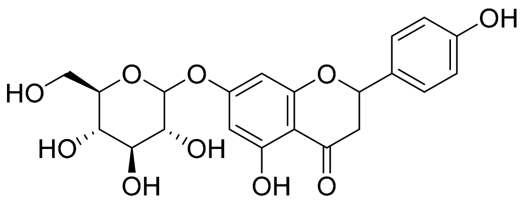

Naringenin-O-glucoside

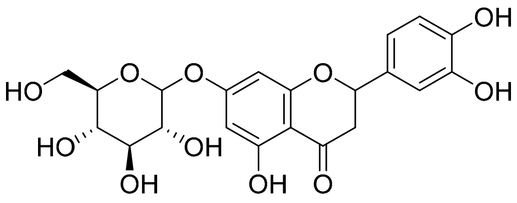

Eriodictyol-O-Glucoside

Figure S1. Chemical structures of the main markers.

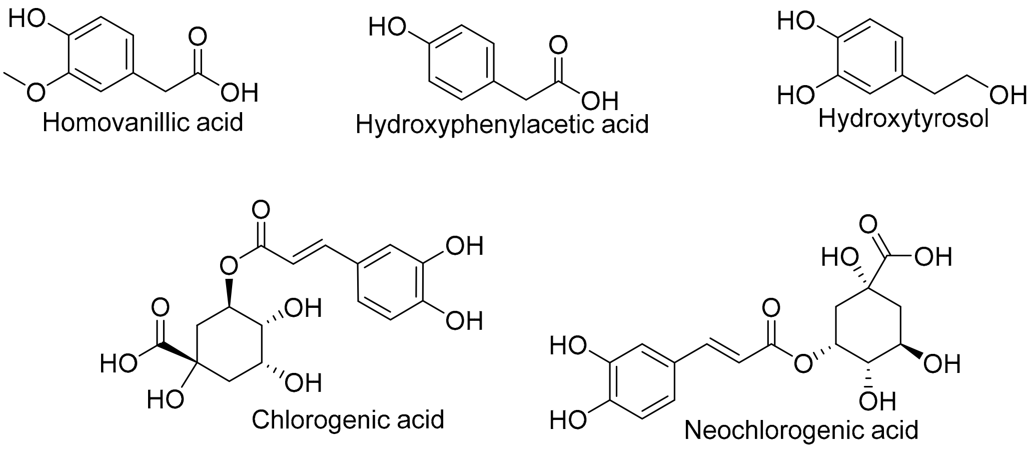

Figure S2. Phenolic compounds showed significant variation between the Pebroter and Roli Rosa varieties.

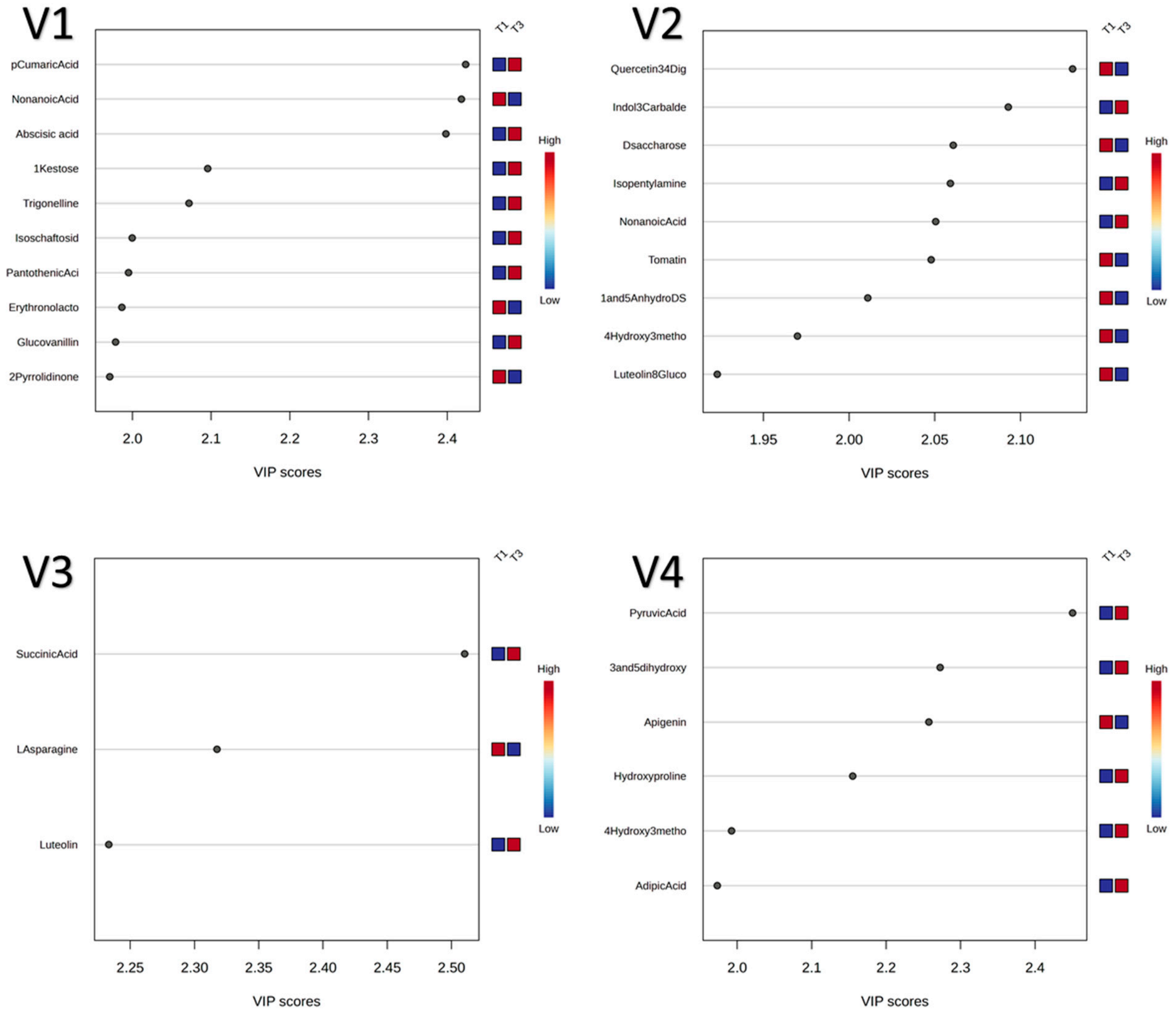

Figure S3. OPLS-DA VIP scores for the selected compounds in T1 versus T3. V1: Cornabel. V2: Cuban pepper. V3: Corno adino. V4: Roli rosa. T1: N-rich compost fertilizer from woody residues. T3: N-poor woody chips fertilizer.

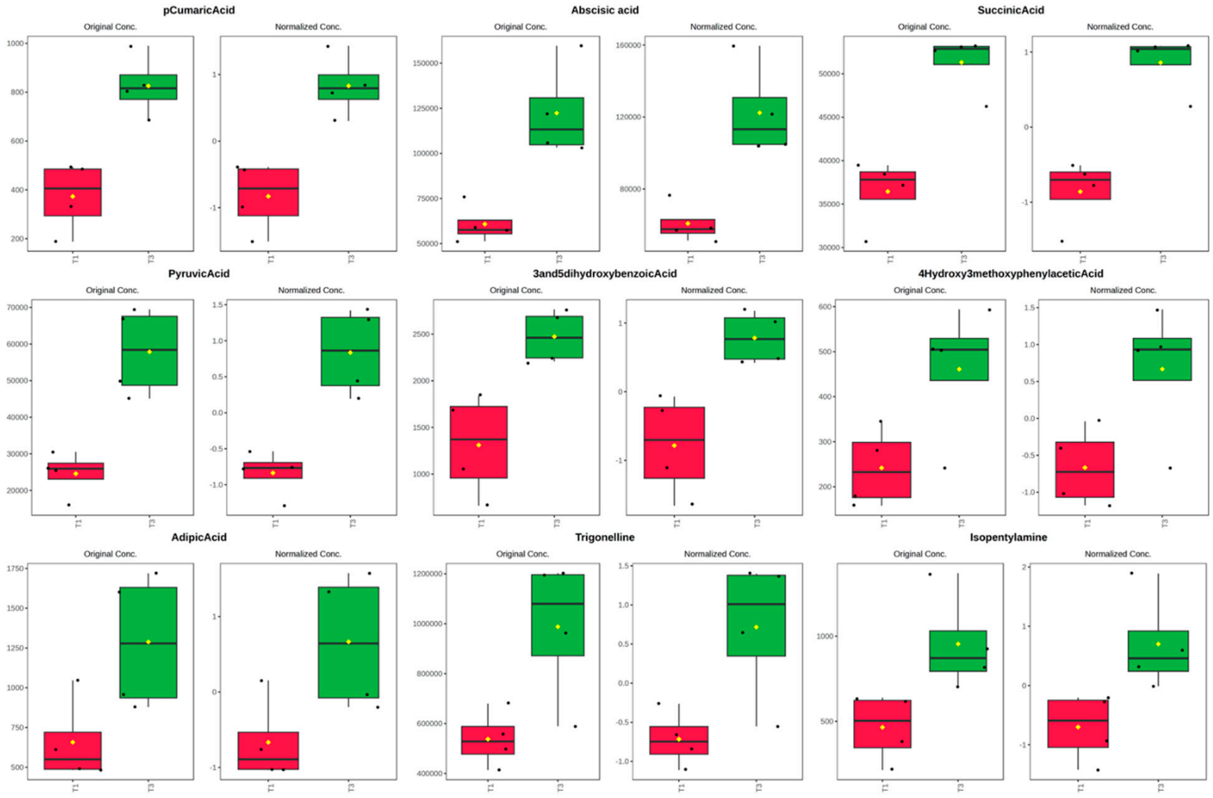

Figure S4. Box-plots of organic acids and amines show variations between T1 versus T3.

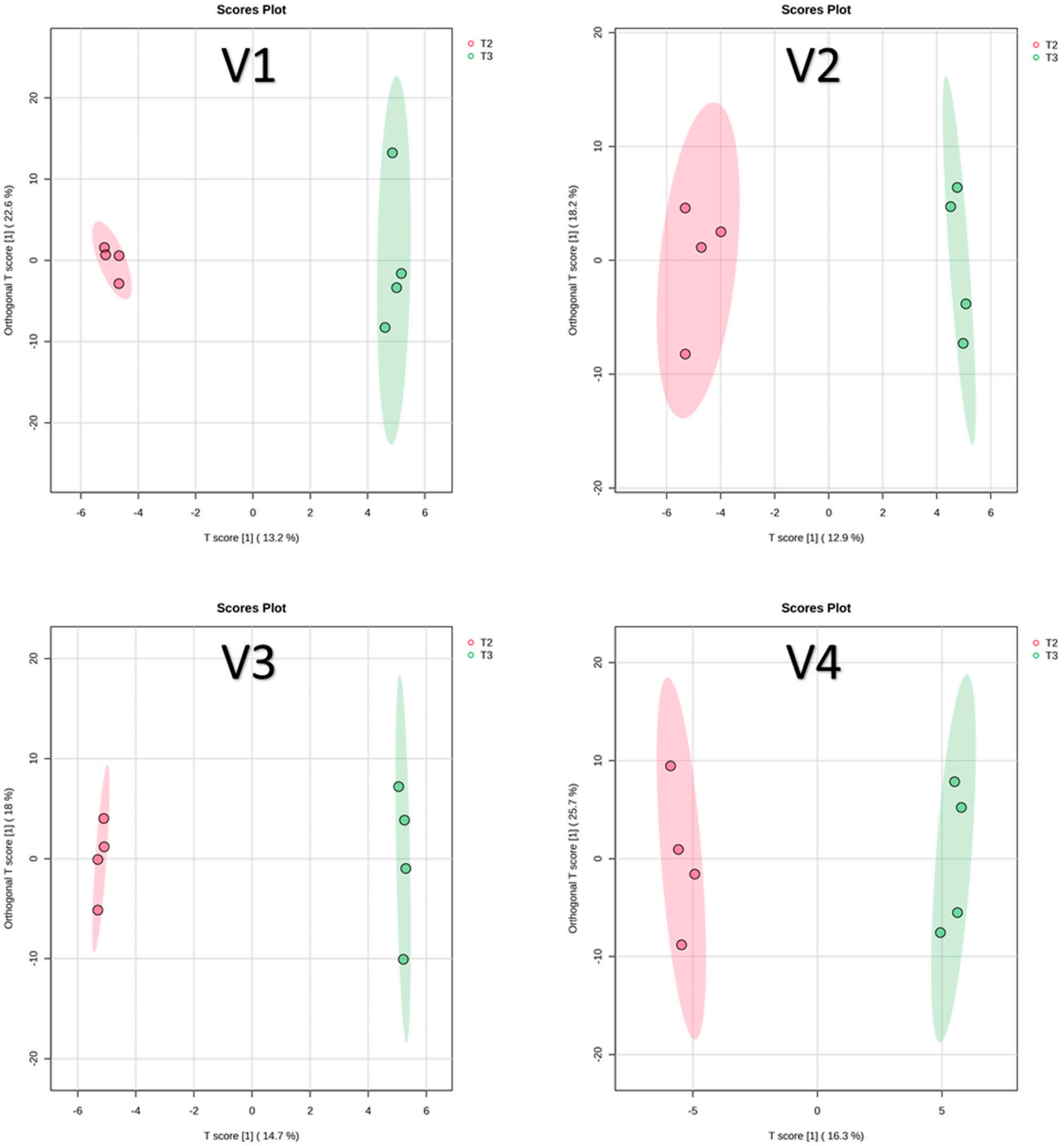

Figure S5. OPLS-DA score plots for T2 versus T3. V1: Cornabel. V2: Cuban Pepper. V3: Corno Adino. V4: Roli Rosa. T2: control. T3: N-poor woody chips fertilizer. n = 4 biological replicates per treatment-variety.

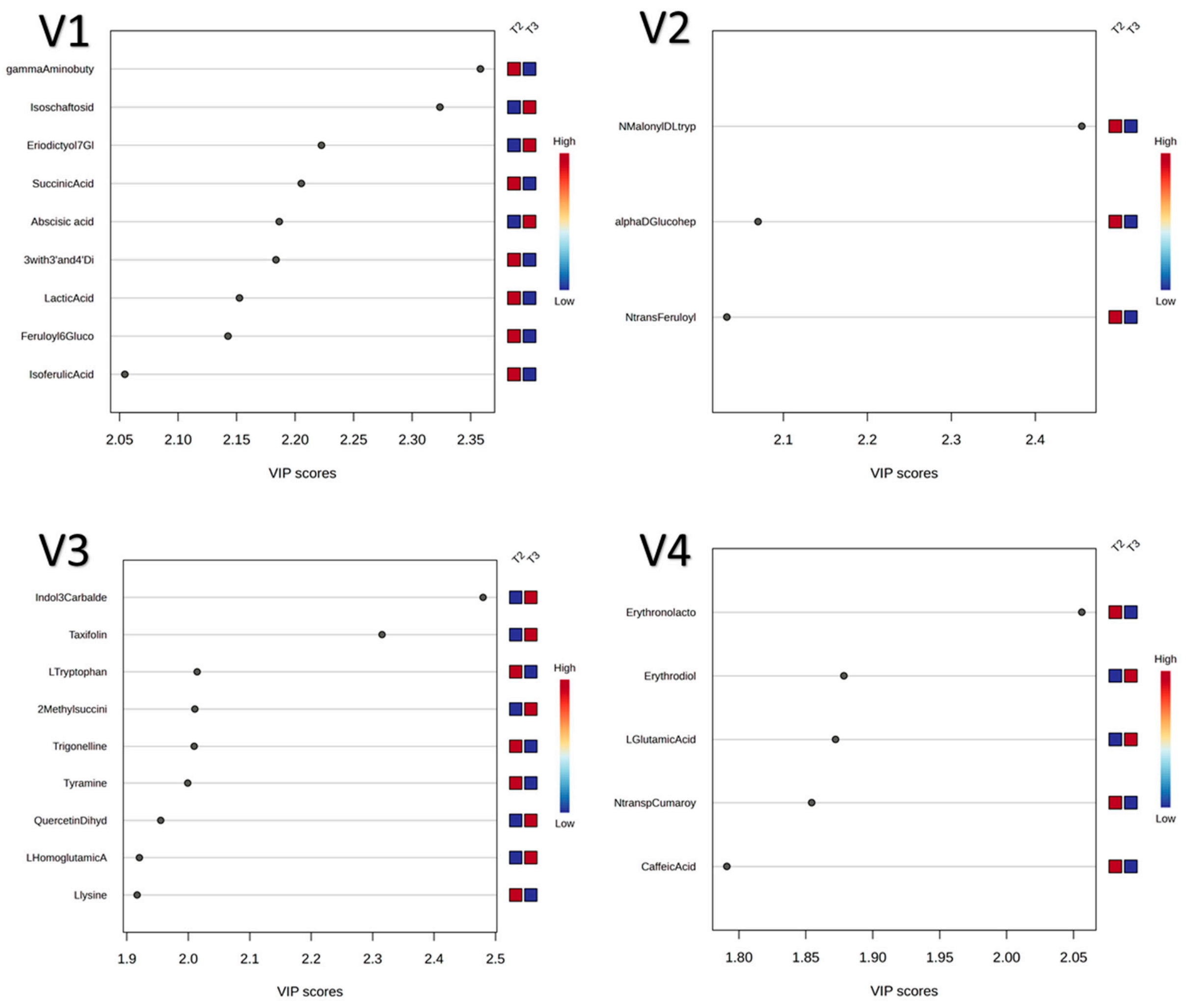

Figure S6. OPLS-DA VIP score for the selected compounds in T2 versus T3. V1: Cornabel. V2: Cuban Pepper. V3: Corno Adino. V4: Roli Rosa. T2: control. T3: N-poor woody chips fertilizer.

Table S1. Internal standards used in *UHPLC-QToF-MS* evaluation of instrument performance.

#	**Internal Standard**	**Full Chemical Name**	**Chemical Class**
1	D-[UL-$^{13}C_6$]Mannit	Uniformly $^{13}C_6$-labeled D-Mannitol	Sugar alcohol (polyol)
2	[UL-$^{13}C_6$-Fru]Sac	Sucrose with uniformly $^{13}C_6$-labeled fructose moiety	Disaccharide
3	[$^{13}C_5$,^{15}N]Glu	L-Glutamic acid-[$^{13}C_5$,^{15}N]	Amino acid (acidic)
4	[$^{13}C_5$,^{15}N]Val	L-Valine-[$^{13}C_5$,^{15}N]	Amino acid (branched-chain)
5	[$^{13}C_9$,^{15}N]Phe	L-Phenylalanine-[$^{13}C_9$,^{15}N]	Amino acid (aromatic)
6	HisAm-d_4	Histamine-d_4	Biogenic amine
7	Me-αD-Glc	Methyl-α-D-glucopyranoside	Methylated monosaccharide
8	D-Pinit	D-Pinitol (3-O-methyl-D-chiro-inositol)	Cyclitol (sugar alcohol derivative)
9	Me-Do-Rib	Methyl-2-deoxy-D-riboside	Deoxysugar
10	Ph-βD-Glc	Phenyl-β-D-glucopyranoside	Aromatic glycoside
11	2Am6Cl-Purin	2-Amino-6-chloropurine	Purine analog
12	t-3,4-MetdiO-CinnA	trans-3,4-Methylenedioxycinnamic acid	Phenylpropanoid (cinnamic acid derivative)
13	5Br2OH-HippuricA	5-Bromo-2-hydroxyhippuric acid	Aromatic acid
14	Benzoic acid-d_5	Benzoic acid-d_5	Aromatic carboxylic acid
15	CholicA-d_4	Cholic acid-d_4	Bile acid (steroid acid)
16	FerulicA-d_3	Ferulic acid-d_3	Hydroxycinnamic acid (phenolic acid)
17	[$^{13}C_6$]tRES	[$^{13}C_6$]-trans-Resveratrol	Stilbene-type polyphenol
18	2Cl-PhEtAm	2-Chlorophenylethylamine	Aromatic amine
